# Coroglaucigenin enhances the radiosensitivity of human lung cancer cells through Nrf2/ROS pathway

**DOI:** 10.18632/oncotarget.16454

**Published:** 2017-03-22

**Authors:** Meng Sun, Dong Pan, Yaxiong Chen, Ya Li, Kun Gao, Burong Hu

**Affiliations:** ^1^ State Key Laboratory of Applied Organic Chemistry, College of Chemistry and Chemical Engineering, Lanzhou University, Lanzhou, 730000, China; ^2^ Key Laboratory of Heavy Ion Radiation Biology and Medicine of Chinese Academy of Sciences, Institute of Modern Physics, Chinese Academy of Sciences, Lanzhou, 730000, China; ^3^ Key Laboratory of Space Radiobiology of Gansu Province, Lanzhou, 730000, China

**Keywords:** coroglaucigenin, Calotropis gigantea, human lung cancer, radiosensitivity, Nrf2/ROS

## Abstract

Seven cardenolides isolated from the ethanol extract of the stems of *Calotropis gigantea* were evaluated *in vitro* against human cancer cells and the structure-activity relationships were discussed. The results demonstrated that a compound, named CGN (coroglaucigenin), had better anti-proliferative activity with the IC_50_ value less than 6 μM among these compounds. Further, we found that CGN displayed much lower cytotoxicity to normal lung epithelial cells (BEAS-2B) than cancer cells (A549). Especially, our results demonstrated that treatment with CGN (1 μM) combined with X-ray irradiation induced higher radiosensitivity in human lung cancer cells (A549, NCI-H460, NCI-H446) but not in BEAS-2B. The expression levels of nuclear transcription factor Nrf2 and Nrf2-driven antioxidant molecule NQO-1 reduced in A549 cells after combined treatment compared to the radiation only. However, CGN had no toxicity and the levels of antioxidant molecules expression were higher in BEAS-2B cells when given the similar treatment as A549 cells. These results suggest that CGN is a very promising potential sensitizer for cancer radiotherapy, which not only inhibits the proliferation of cancer cells but also enhances the radiosensitivity of cancer cells through suppressing the expression of antioxidant molecules while there is no influence for normal cells.

## INTRODUCTION

Lung cancer is one of the most common malignancies worldwide in terms of both incidence and mortality, with 1.3 million new cases being diagnosed more than a million deaths each year. Only 13% of lung cancer patients survive more than 5 years [1]. Radiotherapy is a critical treatment strategy for lung cancer so as to achieve local control and reduce the risk of recurrence [2], with nearly two-thirds of all cancer patients receiving radiotherapy sometime during their illness [3]. However, its curative effect is sometimes limited by radioresistance of the cancer cells as well as radiation toxicity to normal tissue [4]. Thus, finding novel radiosensitizing agents that can increase the radiosensitivity of lung cancer, or which are less toxic to normal tissues have become an area of interest for radiation oncology investigators.

Reactive oxygen species (ROS) are implicated in diverse cellular processes, including cellular metabolic and signaling processes, and they play important roles in a variety of diseases, including cancer. A number of studies have found that overexpression of antioxidant enzyme occurs in cancer resistance to radiation therapy, whereas blocking these antioxidant defenses can enhance radiation sensitivity [5–9]. Nuclear factor-erythroid 2-related factor-2 (Nrf2), a redox sensitive transcription factor, regulates the expression of several detoxifying enzymes, such as heme oxygenase-1 (HO-1), and nicotinamide adenine dinucleotide phosphate (reduced) (NADPH): quinone oxidoreductase-1 (NQO-1), through binding to antioxidant response element (ARE) within gene promoters [9, 10]. Ionizing radiation (IR) causes DNA nucleotide modifications, single and double strand DNA breaks (SSBs and DSBs), both directly and indirectly via formation of free radicals and ROS. It is generally estimated that approximately two-thirds of IR-induced DNA damage is caused by the latter [11, 12]. Cells have therefore developed efficient systems for maintaining their genomic integrity, such as the DNA damage response (DDR). Phosphorylated H2AX (γH2AX) acts as a signal for DNA damage which promotes conformational rearrangements in the chromatin near DSB sites, leading to rapid recruitment of DDR proteins [12, 13]. Using of the specific antibodies, γH2AX foci can be observed at the DSB sites induced by irradiation and have been reported as a good surrogate marker of DSBs [14, 15]. The γH2AX foci assay not only has been suggested as a reliable tool to study DSB induction and repair [16] but also used to evaluated the DNA damage induced by toxicity reagent including IR.

Cardiac glycosides (to which belong a class of drugs named cardenolides) have long been used in the treatment of heart failure. The biological functions of cardenolides are explained by inhibition of Na^+^/K^+^-ATPase leading to an increase of intracellular Ca^2+^, which results in a better interaction between actin and myosin filaments in cardiac myocytes [17]. Cardenolides have also been paying noticeable attention with respect to their potential use as anticancer agents [18]. However, there is little coverage on their radiosensitivity roles and the underlying mechanism. With the aim to investigate these effects, ten known cardenolides named (19S)-3*β*,19-epoxy-2*α*,3*β*,14*β*-trihydroxy-19-methoxy-5*α*-card-20(22)-enolide (1) [19], uzarigenin (2) [20], digitoxigenin (3) [21], corotoxigenin (4) [22], calotropagenin (5) [23], coroglaucigenin (6) [24], gomphoside (7) [25], calotropin (8) [25], calactin (9) [25], 6′-*O*-(*E*-3,5-dimethoxy-4-hydroxycinnamoyl) desglucouzarin (10) [26] were isolated from the stems and leaves of *Calotropis gigantea* from Hainan Province in China. The anti-proliferative activities of the isolated compounds 1, 2, 5, 6, 8-10 on A549, HeLa and 786-O cell lines were evaluated by a cytotoxic MTT assay. We found that compound 6 (CGN) showed better suppressing proliferation ability on A549 cells but slighter toxicity to human normal lung epithelial cells (BEAS-2B). Colony formation assay showed that CGN enhanced the radiosensitivity of lung cancer cell lines A549, NCI-H460, NCI-H446. Furthermore, the mechanisms underlying the CGN enhancing the radiosensitivity to A549 cancer cells and protecting the normal BEAS-2B cells were investigated.

## RESULTS

### Structural characteristic and initial screening

**Figure 1:**
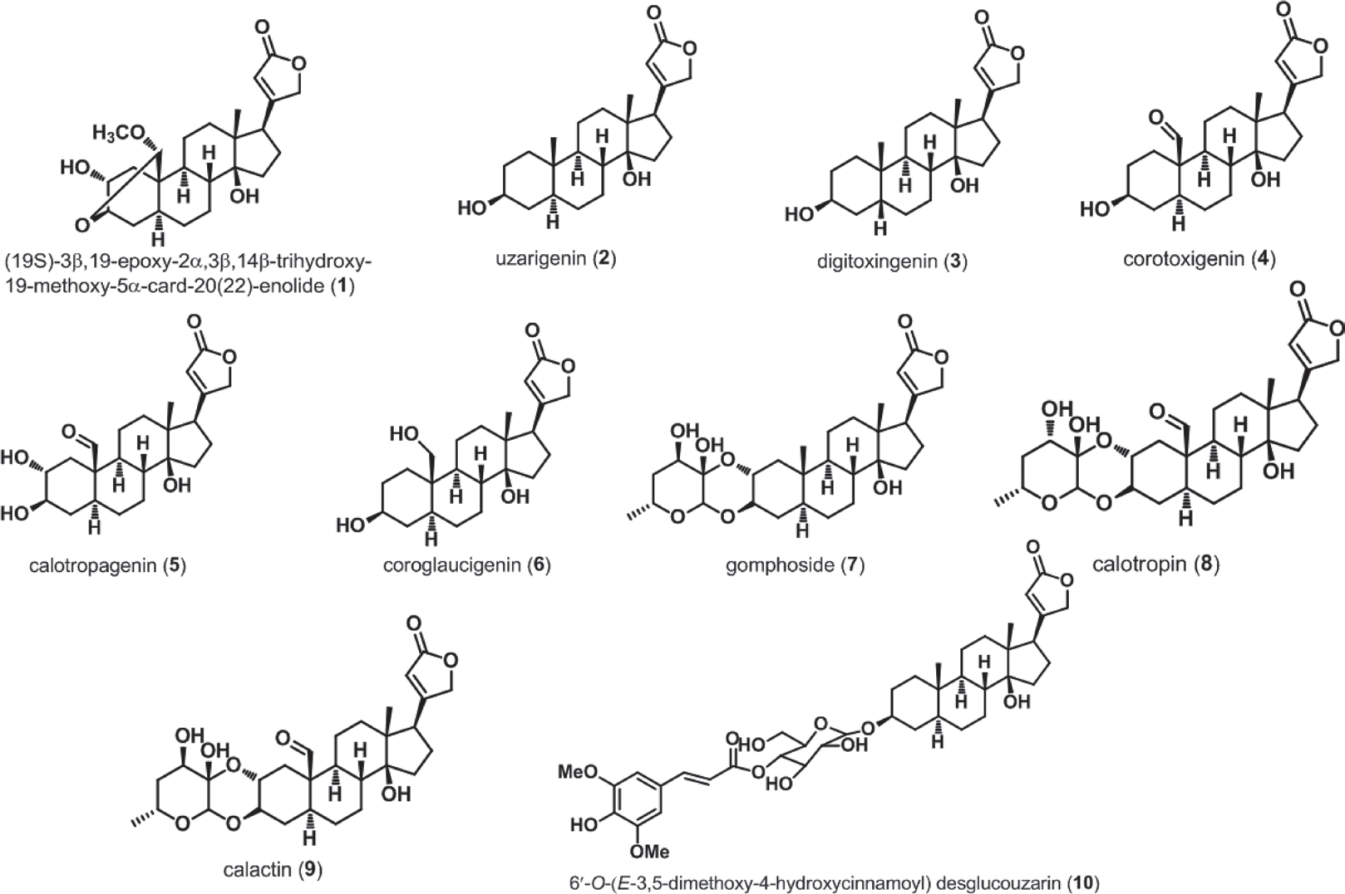
Chemical structures of cardenolides isolated from *Calotropis gigantea* Ten cardenolides were isolated from *C. gigantea*. Their structures were determined on the basis of spectral data.

Ten cardenolides (compounds 1-10), the chemical constituents of the active antitumor fractions, were obtained by means of chromatographic separation and their structures were determined on the basis of spectral data. As shown in Figure 1, cardenolide is a special constituent of steroid containing certain structural differences such as *cis* A/B and C/D ring junctions, a tertiary hydroxyl group at C-14 and a butenolide substituent at C-17. Compound 2, a methyl group locating at C-10, is designated as the basic structure of cardenolides. Compound 3 is a diastereomer of 2. The replacement of a formyl or a hydroxymethyl at the C-10 position of compound 2 leads to compounds 4 or 6, respectively. Compound 2 introduced an α-hydroxyl group at C-2 position generates compound 5. Compound 1 is formed by the intramolecular acetal formation of compound 5 involving *β*-positioned hydroxy group at C-3 and the formyl group at C-19. Compounds 7-9 are doubly linked cardenolide glycosides in which compound 8 is a diastereomer of 9. Compound 10 is linked to a mono 6-deoxyhexose moiety.

The anti-proliferative activities of the isolated compounds 1, 2, 5, 6, 8-10 except for compounds 3, 4 and 7 (their amounts are not enough for activity investigation) on A549, HeLa and 786-O cells were evaluated by a cytotoxic MTT assay. The results are summarized in Table 1. Most cardenolides showed cytotoxic activity against these three kinds of cancer cell lines, with the exception of compounds 5 and 10. An activity ranking reveals that compound 9 > compound 8 > CGN (compound 6) > compound 2 > compound 1 > compound 5 > compound 10. These results highlight the importance of the substituent group at C-10 position and the doubly linked cardenolide glycosides (8 and 9) have stronger cytotoxicity than those of the cardenolides (1, 2, 5 and CGN). On the basis of these results, we selected our “lead” compounds, CGN and compound 8, for the further radiosensitivity and underlying mechanism experiments.

**Table 1:** *In vitro* cytotoxic activity represented as IC_5__0_ values (μM) on A549, HeLa and 786-O cell lines of seven compounds isolated from *Calotropis gigantea* were measured by MTT assays

Compounds	IC_50_ (μM, A549)	IC_50_ (μM, HeLa)	IC_50_ (μM, 786-O)
1	16.5 ± 2.9	13.3 ± 2.2	20.0 ±1.4
2	12.0 ± 0.6	5.2 ± 1.4	14.2 ± 0.8
5	> 25	> 25	> 25
6	4.6 ± 1.2	4.3 ± 0.9	5.3 ± 1.6
8	4.2 ± 0.7	3.7 ± 1.0	6.0 ± 2.1
9	2.2 ± 1.2	1.2 ± 0.2	3.2 ± 0.7
10	> 25	> 25	> 25

### CGN enhances the killing capability of irradiation on human lung cancer cells while not on normal epithelial cells

**Figure 2:**
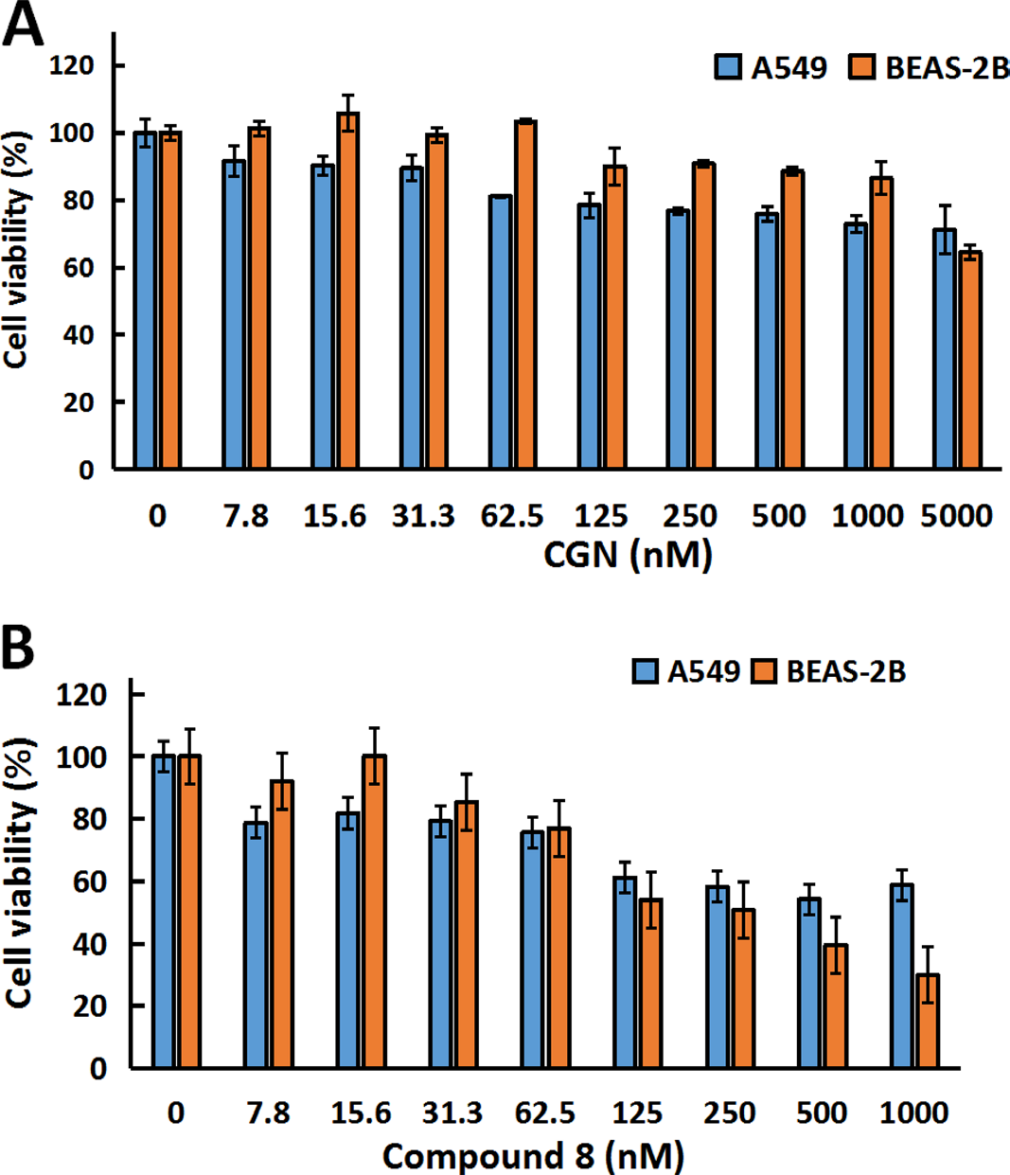
CGN inhibits proliferation of cancer cells but not for normal epithelial cells Cytotoxicity screening of CGN (**A**) and compound 8 (**B**) in A549 and BESA-2B cells. Cells (5×10^3^ cells/well) were plated in 96-well plates for 24h and subsequently treated with varying concentrations of drugs, and the cell viability was determined by MTT assay.

**Figure 3:**
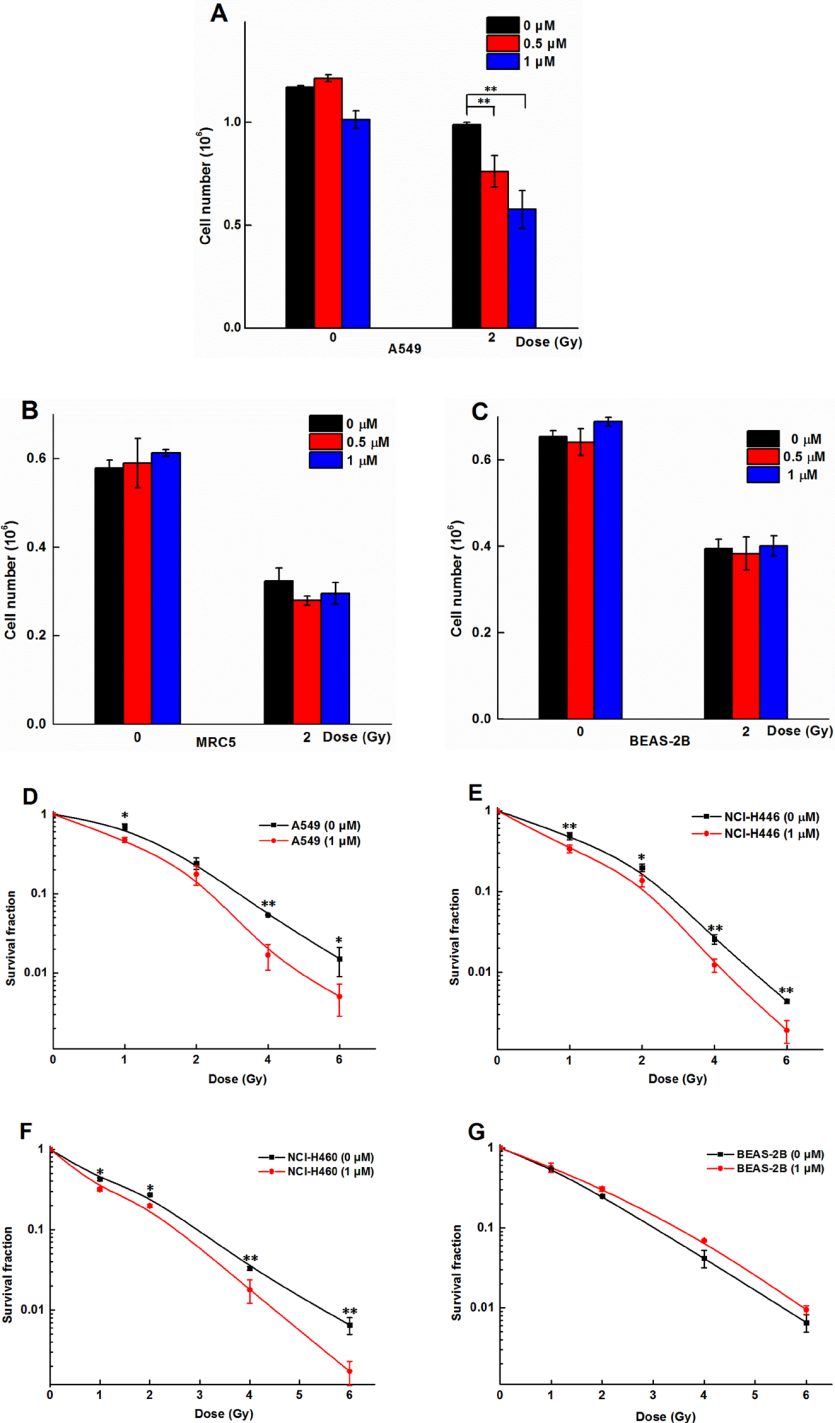
CGN enhances the killing capability of irradiation on cancer cells but not on normal fibroblast and epithelial cells Graphic representation of cell number of A549 (**A**), MRC5 (**B**) and BEAS-2B (**C**). Cells after CGN pretreatment combined with or without X-ray irradiation. Cell numbers were scored by Z1 Cell Counter (Beckman). Survival fractions of A549, NCI-H446, NCI-H460 and BEAS-2B cells pretreated with or without CGN (1 μM) followed by 0, 1, 2, 4 and 6 Gy of X-ray irradiation were measured by colony score (**D**–**G**). Data are presented as means ± SD (*n* = 3). *P < 0.05; **P < 0.01 *vs*. the irradiation only group.

Figure 2A and 2B showed that compound 8 had cytotoxicity against both A549 and BEAS-2B. While treatment of the cells with CGN (≤ 1 μM) had no cytotoxicity on BEAS-2B cells, compared with the A549 cells. Thus, CGN is more effective and less toxic than 8 for treating lung cancer and the concentrations of 0.5 and 1 μM were chosen to study the synergistic or adverse effect of cardenolide on radiation. Figure 3A showed that cell number of A549 cells decreased by 15.6 ± 1.3% for radiation treatment at 2 Gy alone. When the cells were pretreated with 0.5 or 1 μM CGN and then irradiated with 2 Gy X-rays, the cell number decreased by 26.1 ± 6.1% (0.5 μM) and 45.0 ± 6.8% (1 μM) compared with the X-ray irradiation alone (set as 100%) (Figure 3A). However, there were no significant toxicity for MRC5 (Figure 3B) and BEAS-2B (Figure 3C) cells after the combined treatment. To further investigate the relationship between the CGN treatment and the radiosensitivity, human lung cancer cells were pretreated with CGN (0, 1 μM) for 24 h and then irradiated with 0, 1, 2, 4, 6 Gy of X-rays. As illustrated in Figure 3D–3F and Supplementary Table 1 CGN enhanced the radiosensitivity of all cancer cell lines, especially at the doses of 4 and 6 Gy. However, CGN had a radio-protective effect on BEAS-2B cells (Figure 3C and 3G). These results suggest that CGN is a putative radiosensitizer for lung cancer cells, meanwhile, it has low side-effect of radiation on human normal lung cells such as normal fibroblast and epithelia. The above results also indicate that the drug concentrations at 0.5 and 1 μM are better choices for further experimental studies.

### DNA damage was enhanced after CGN pretreatment in A549 cells

**Figure 4:**
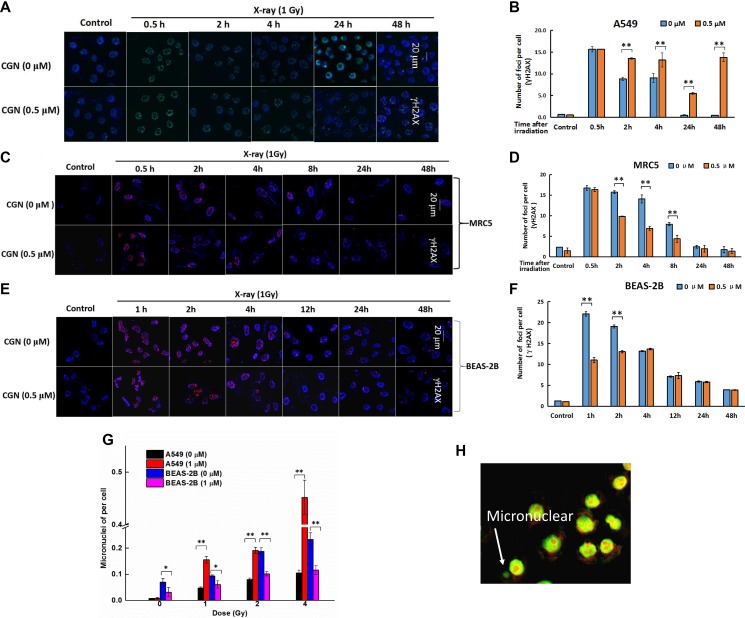
DNA damage was enchanced after CGN pretreatment in A549 cells Effect of CGN pretreatment combined with X-ray irradiation on the DNA damage of lung cancer cells. Nuclear staining was done with DAPI (blue) and γH2AX staining appeared as red points (foci). Scale bars represent 20 μm. Effect of CGN pretreatment combined with X-ray irradiation on DNA damage of A549 (**A**, **B**), MRC5 (**C**, **D**) and BEAS-2B cells (**E**, **F**). Cytotoxicity of CGN and/or X-rays to cells was indicated by micronuclei. Five hundred A549 or BEAS-2B cells were scored under microscopy to determine the frequency of cell with micronuclei (**G**). Structures that were morphologically identical to but smaller than the cell nucleus were considered to be micronuclei and their diameter should not be larger than one third of the diameter of the cell nucleus. The typical image of micronuclei was presented (**H**). **P < 0.01 *vs*. non drug-treated cells.

More γH2AX foci was observed in A549 cells after pretreatment with 0.5 μM CGN followed by X-ray irradiation (Figure 4A, 4B), compared to the X-ray irradiation alone. X-ray induced γH2AX gradually decreased in a time-dependent manner (Figure 4A, 4B), however, the speed of the decrease of DNA damage foci was slower and kept in a higher fraction for the combined treatment. At 24 and 48 h time point, higher numbers of foci in cells after the combined treatment were observed to increase by 10- and 27-folds than the X-ray irradiation alone (Figure 4B). In addition, the fluorescent intensity of γH2AX foci was also significantly higher at both 24 and 48 h after X-rays + CGN treatment compared with the X-rays alone. We further investigated whether CGN could enhance genotoxicity on normal lung cells induced by irradiation. As shown in Figure 4C–4F, CGN did not increase the fraction of γH2AX foci in normal lung cells (BEAS-2B and MRC5 cells) after irradiation. These results suggest that pretreatment with CGN can enhance DNA damage induced by X-rays in cancer cells but not in normal lung cells.

During mitotic exit, missegregated chromosomes can recruit their own nuclear envelope to form micronuclei [27]. Micronucleus are a good surrogate marker of chromosome instability and also closely related to DSBs induction [28]. The effect of CGN on X-ray irradiation mediated genotoxicity and cytotoxicity was evaluated using the micronucleus assay and the typical image of micronuclei was presented in Figure 4H. There was no significant increase in the number of cells with micronuclei for the CGN-alone-treated A549 cells compared with its 0.001% of DMSO treated control cells (Figure 4G). Pretreated A549 cells with 1 μM CGN followed by different doses of X-rays led to the increase in the fraction of cells with micronuclei in a dose-dependent manner (Figure 4G). However, CGN treatment did not increase the fraction of cells with micronuclei in BEAS-2B at any dose of X-rays when compared with the corresponding dose of irradiation alone (Figure 4G). Our micronucleus assay also suggests that pretreatment with CGN enhances the radiosensitivity of lung cancer cells. Taken together, we consider that CGN only enhances the damage of DNA of cancer cells but not the normal lung epithelia under the experimental concentration in this study.

### CGN treatment aggravates the oxidative stress and oxidative damage induced by radiation

**Figure 5:**
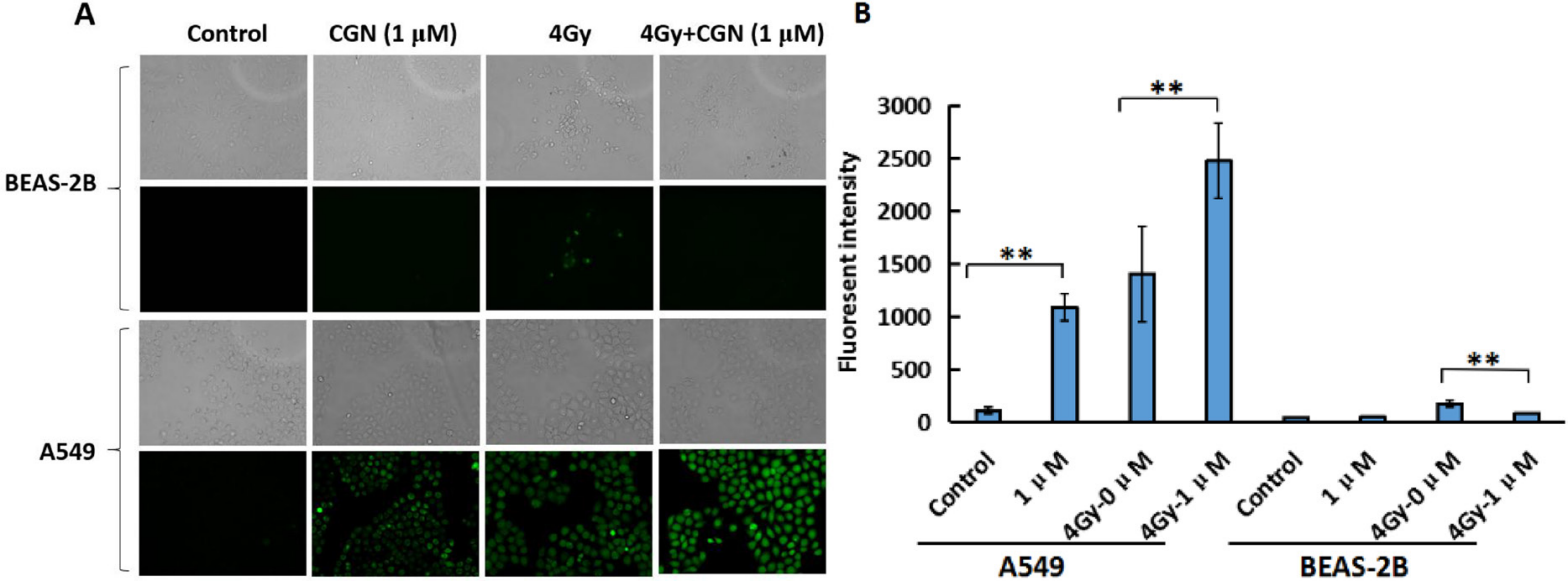
CGN treatment aggravates the oxidative stress and oxidative damage induced by radiation in A549 cells The exponential grown BEAS-2B (upper) and A549 (lower) cells were pretreated with CGN for 24 h and then followed by X-ray irradiation. The intracellular ROS levels were determined by means of fluorescent intensity of DCFH-DA-stained IECs assay. The images are shown as the representative from three independent experiments. The upper shows the images taken under the bright phase-contrast microscope and the lower shows the images taken under the fluorescent microscope (×10) in each group (**A**). Fluorescence intensity were quantified using ImageJ software (**B**). One hundred cells were quantified to determine the average fluorescence intensity. **P < 0.01 *vs*. X-ray irradiation alone.

Oxidative stress is the major mechanism for radiation-induced cancer cell death. ROS formation was examined using cells loaded with DCFH-DA, a dye that is oxidised into a highly fluorescent form in the presence of peroxides. As shown in Figure 5A and 5B, ROS generation significantly increased in A549 cells after treatment with CGN whereas, there was no obvious change in the generation of ROS in BEAS-2B cells after the same treatment compared with their control groups. The ROS formation in BEAS-2B cells significantly reduced after the combined treatment when compared with the X-rays alone. CGN pretreatment might increase the cellular ROS level in A549 cells, which thereby promotes the killing ability to cancer cells.

### CGN pretreatment enhances the G2/M phase arrest induced by irradiation

**Figure 6:**
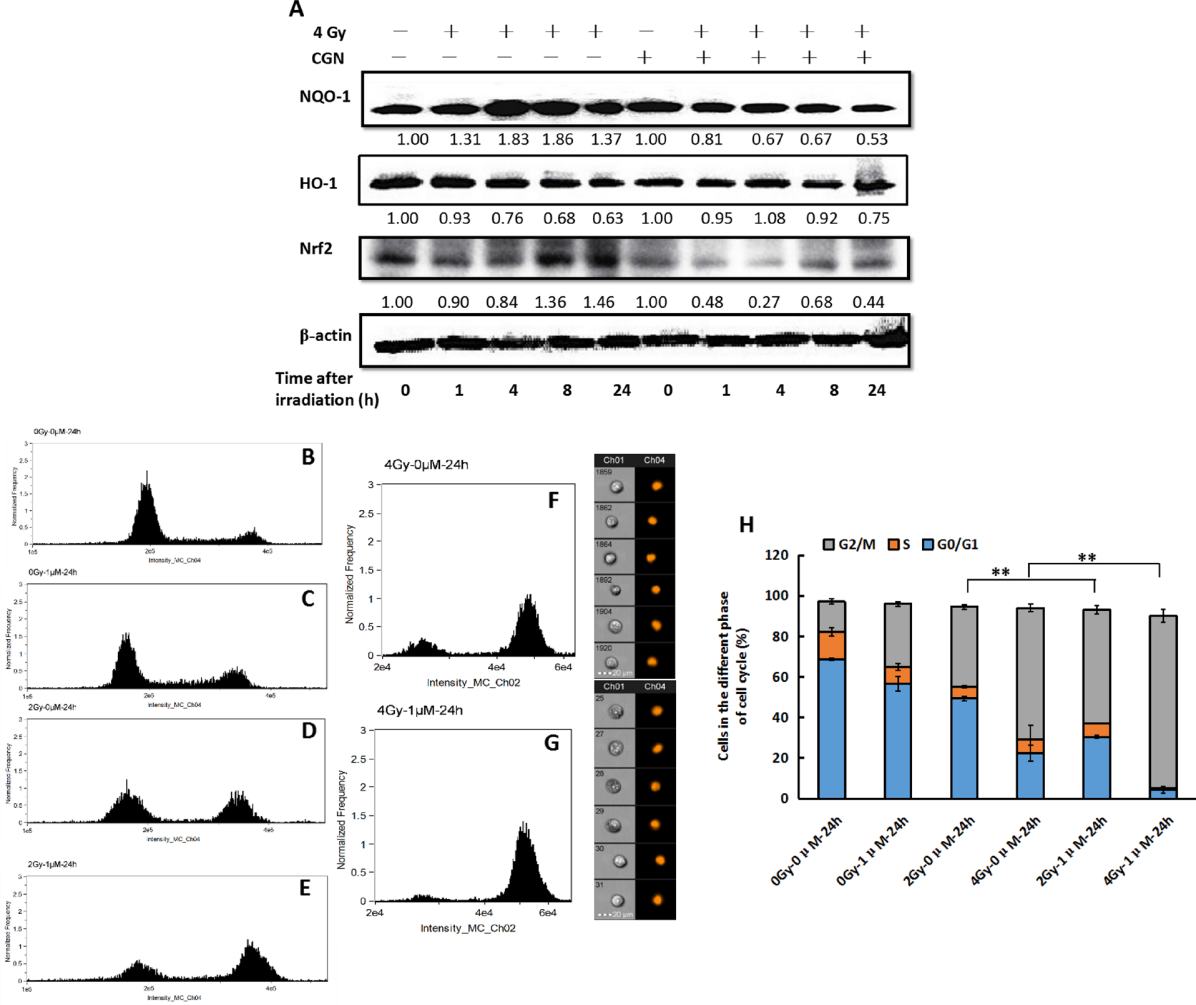
CGN pretreatment enhances the G2/M phase arrest induced by irradiation The cells were harvested at different time point after irradiation. The expressions of proteins were analyzed by western blotting (**A**). Effect of CGN pretreatment combined with X-ray irradiation on cell cycle distribution in A549 cells (**B**–**H**). B, cells treated with 0 μM CGN and 0 Gy X-rays. C, cells treated with 1 μM CGN and 0 Gy X-rays. D, cells treated with 0 μM CGN and 2 Gy X-rays. E, cells treated with 1 μM CGN and 2 Gy X-rays. F, cells treated with 0 μM CGN and 4 Gy X-rays. G, cells treated with 1 μM CGN and 4 Gy X-rays.**P < 0.01 *vs*. X-ray irradiation alone. All experiments were repeated independently for three times and the typical images were presented.

As a global regulator of cellular antioxidant responses, Nrf2 regulates the expression of several detoxifying enzymes including NQO-1 and HO-1 and controls a majority of antioxidant pathways. Thus, we first determined the expression of Nrf2, NQO-1 and HO-1 in A549 cells at 0, 1, 4, 8 and 24 h after treatment with CGN. Protein levels of NQO-1 and HO-1 decreased obviously at 24 h. Significant decline in Nrf2 level was first observed at 4 and 8 h, and then the expression of Nrf2 returned to normal level. However, the expression of Nrf2 decreased again 24 h later (Figure 6A). It is generally accepted that ROS eventually cause DNA damage. IR can induce the activation of cell cycle checkpoint proteins to arrest cell cycle progress. Subsequently, we evaluated the effects of X-rays combine with (Figure 6E and 6G) or without CGN (Figure 6D and 6F) on cell cycle. As shown in Figure 6H, the percentage of A549 cells at G2/M phase were 39.5 ± 1.1% (Figure 6D) and 64.8 ± 1.9% (Figure 6F) after the cells were irradiated with 2 and 4 Gy, respectively (the percentage of G2/M phase are 14.9 ± 1.4% (Figure 6B) in the untreated control cells and 31.2 ± 1.1% (Figure 6C) in the drug-treated only cells. A higher level of G2/M phase arrest was observed after pretreated A549 cells with 1 μM CGN followed by irradiation and then incubation for 24 h. The fractions of cells at G2/M phase increased to 55.9 ± 2.1% at 2 Gy (Figure 6E) and 83.1 ± 3.2% at 4 Gy (Figure 6G). Therefore, the G2/M phase arrest may in part account for the effect of CGN on the enhancement of radiosensitivity of A549 cells.

### Down-regulation of the antioxidant defense system in cancer cells and up-regulation in normal cells after pretreatment with CGN

**Figure 7:**
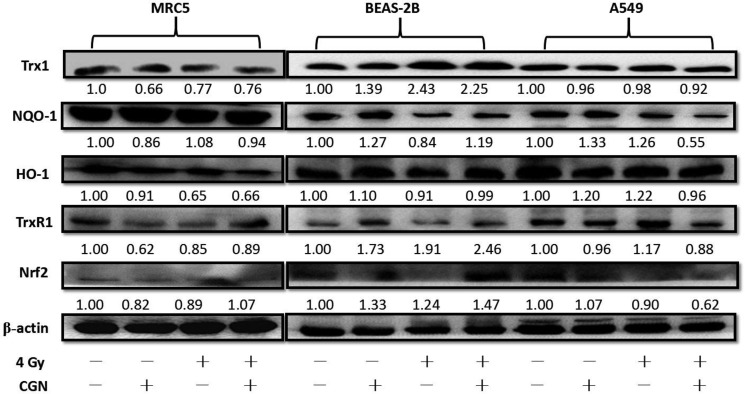
Down-regulation of the antioxidant defense system in cancer cells and up-regulation in normal cells after pretreatment with CGN and/or irradiation The antioxidant molecules expression were measured by western blotting in normal cells (MRC5 and BEAS-2B) and cancer cells (A549) at 24 h after pretreatment with or without 1 μM drugs for 24 h and then followed by 0 or 4 Gy X-ray irradiation. The cells (1×10^6^ cells/dish) were seeded in 60 mm dishes for 24 h, followed by CGN treatment for another 24 h and then exposed to 4 Gy X-rays. The cells were harvested at 24 h post-irradiation. The expressions of proteins were analyzed by western blotting. All experiments were repeated independently for three times and the typical images were presented.

The differences in the radiosensitivity and ROS formation observed between BEAS-2B and A549 cells led us to speculate the differences of their redox-maintaining mechanisms. The expression level of the antioxidant molecules in cells were altered after treatment with CGN at nontoxic concentration (1 μM) followed by irradiation with 4 Gy X-rays (Figure 7). NQO-1 and TrxR1 reduced in CGN pretreated cancer cells (A549) compared with the X-rays only. Intriguingly, these two proteins’ expressions elevated in BEAS-2B cells and the level of expression of NQO-1 did not show obvious change in MRC5 cells after combined treatment compared with the radiation alone, which may protect the normal cells from irradiation induced DNA damage. The expression of Nrf2 in A549, BEAS-2B and MRC5 cells were also measured using western blotting. The expression of Nrf2 in the combined treatment obviously increased in BEAS-2B cells (Figure 7) and slightly increased in MRC5 cells (Figure 7) compared to their irradiation only, while the expression of Nrf2 in A549 cells was lower in the combined treatment compared to the irradiated only (Figure 7). Our data suggest that pretreatment of cells with CGN followed by irradiation with X-rays effectively inhibits the transcription of Nrf2-driven antioxidant genes in A549 cells.

### Requirement of Nrf2 for the radiosensitivity of CGN

**Figure 8:**
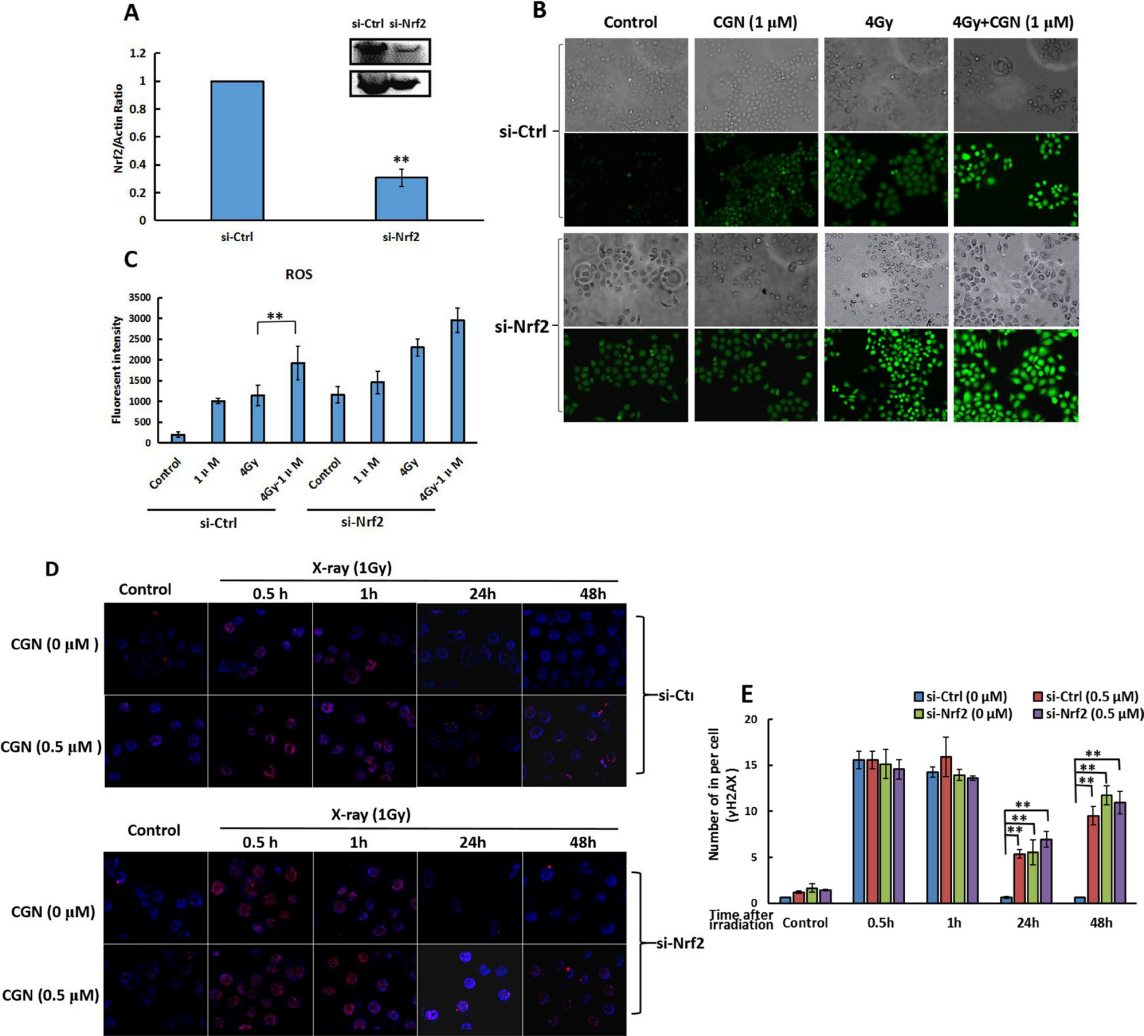
Expression of Nrf2 affects the radiosensitivity mediated by CGN Nrf2 expression in cells transfected by si-Nrf2 and si-Ctrl were determined by western blot (**A**). Si-Ctrl or si-Nrf2 transfected cells were pretreated with CGN for 24 h and then irradiated. The levels of ROS in cells were detected by immunofluorescence microscopy after staining with DCFH-DA (**B**). Fluorescence intensity were quantified using ImageJ software (**C**). The induced γH2AX foci in si-Ctrl or si-Nrf2 transfected cells were measured after pretreatment with CGN for 24 h and then exposure to X-rays (**D**, **E**). **P < 0.01 *vs*. untreated control group.

We further explored the role of Nrf2 for the radiosensitivity of CGN in response to X-rays. We transfected the cells with the siRNA specifically targeting the Nrf2 to generate the A549 cells silencing the expression of Nrf2 (si-Nrf2). As a control, A549 cells were also transfected with a nontargeting siRNA to afford the control cells (si-Ctrl). After confirming the knockdown efficiency of Nrf2 by western blot analysis (Figure 8A), we evaluated the effect of CGN against X-ray induced oxidative challenge toward different cells. As shown in Figure 8B and 8C, irradiation did not significantly increase the level of ROS in si-Nrf2 cells pretreated with CGN compared to no CGN. The increase of ROS level in si-Nrf2 transfected cells after treatment both with and without CGN + X-rays were similar to the level of ROS in si-Ctrl transfected cells after CGN + X-rays. Likewise, Nrf2 knockdown cells failed to increase the formation of γH2AX foci after pretreatment with CGN and then exposure to X-rays compared to no CGN (Figure 8D and 8E). These results suggest that pretreatment with CGN increases the radiation induced DNA damage in cancer cells involving in an Nrf2-dependent pathway. CGN pretreatment suppresses the expression of antioxidant molecules resulting in a sustained oxidative DNA damage.

## DISCUSSION

Because cancers develop phenotypes that are treatment resistant or treatments causing unwanted and/or detrimental side effects to normal cells or to untargeted tissues, the majority of anticancer therapies fail. NSCLC poses a significant problem in patients because of its resistance to radiotherapy. Hence, treatment strategies that can improve radiosensitivity of NSCLC cells can provide immense benefits and could reduce morbidity among patients. An attractive strategy that can used to enhance the benefits of IR is to find novel radiosensitizing agents which can increase the radiosensitivity of lung cancers and, meanwhile, are less toxic to normal tissues.

In our continuing search for the biologically active natural products, the EtOAc fraction of an EtOH crude extract from *C. gigantea* was found to have significant anti-proliferative activity against the A549 lung cancer cell line in our preliminary experiments. Subsequent experiments showed that the survival rate against A549 cell line of the 50% EtOH/H_2_O subfraction (gained from EtOAc fraction) was < 20% (at 300 μg/mL), and it was thus selected for bioassay-guided fractionation. Ten cardenolides were isolated from the subfraction. The anti-proliferative activities of the isolated compounds 1, 2, 5, 6, 8-10 and the structural characteristics imply the following structure-activity relationships: Firstly, the 3*β*,14*β*-dihydroxyl and a butenolide functionalities proved to be important for the activity. Secondly, an activity ranking (compound 9 > compound 8 > CGN > compound 2 > compound 1 > compound 5 > compound 10) reveals that the importance of the substituent group at C-10 position. These results suggest that the presence of a formyl function, or to a lesser extent, a primary alcohol at position C-10, plays an important role in the cytotoxic activity. It also indicates that the C-19 and C-3 acetal bridge can significantly decrease the cytotoxic activity. Thirdly, compound 10 was inactive, which indicates that a huge substituting group at the sugar moiety can decrease the cytotoxic activity. Finally, as indicated by the IC_50_ values, the doubly linked cardenolide glycosides (8 and 9) have stronger cytotoxicity against A549, 786-O and HeLa cells than those of the cardenolides (1, 2, 5 and CGN). Moreover, calactin (9) is the most potent, whereas its 3′-epimer, calotropin (8), is less potent, indicating the importance of the stereochemistry at C-3′ of the deoxy sugar residue. Taken together, the anti-proliferative activity of these cardenolides and cardenolide glycosides depends on not only the substituent groups at C-10 position, but also the sugar moieties at C-3 position.

Other cardiac glycosides such as digitalis, digitoxin, bufalin and oleandrin have been paid increased attention with respect to their potential use as anticancer agents. Certain cardiac glycosides were reported to enhance the response of tumor cells to irradiation. Ouabain was reported to enhance the radiosensitivity of A549 cells but not the normal human lung fibroblasts [29]. Subsequently, ouabain was shown to radiosensitize human tumor cells of different histology types including squamous cell carcinoma and melanoma [30]. Stenkvist noted that breast carcinoma patients who were on digitalis medication at the time of cancer diagnosis had significantly better response to anticancer therapy and better overall survival than breast cancer patients who were not taking digitalis [31]. Although studies have reported that cardenolides have radiosensitizing effects on cancer cells, the potential mechanisms are still not fully unclear. Our current study not only confirms cardenolide-induced radiosensitization in human lung cancer cells (A549) but also shows that the radiosensitizing effect is not on the normal lung fibroblast (MRC5) or epithelial cells (BEAS-2B).

Radiotherapy and chemotherapeutic agents can effectively kill cancer cells through generation of ROS. Overexpression of antioxidant enzymes in cancer cells can contribute to their resistance to some chemotherapeutic agents and radiotherapy [32]. CGN pretreatment followed by X-ray irradiation exhibited the radioresistence and lower ROS levels in BEAS-2B cells than X-ray irradiation only. While in A549 cells the combined treatment showed higher ROS levels than X-rays only. Thus, we examined antioxidant molecules expression in BEAS-2B (normal bronchial epithelial), MRC5 (human normal lung fibroblast) and A549 (lung adenocarcinoma pulmonary epithelial) cells. Pretreating the normal cells with CGN led to a reduction of the accumulation of ROS and thus may confer the protection against oxidative insults to the BEAS-2B and MRC5 by irradiation. Pretreatment of A549 cells with CGN followed by X-rays displayed lower antioxidant molecules (NQO-1, TrxR1) expression than irradiation only (Figure 8). CGN along with X-rays can inhibit the total protein expression level of Nrf2 in lung cancer cells. This is the first report to show that cardenolide decreases radiation-induced antioxidant molecules in lung cancer cells but increases in the normal lung cells. The phenomena of the opposite effects in BEAS-2B and MRC5 cells indicate that the antioxidant molecules may play an important role in determining the radiosensitivity. The down-regulation of antioxidant molecules in CGN pretreated A549 cells will benefit significantly to the radiotherapy in a majority of lung cancer patients because CGN only slightly inhibit normal lung cell proliferation.

Bindra and Herzon's report evinced that cardiac glycosides are potent inhibitors of DNA DSBs repair [33]. Our data manifest that CGN can enhance DNA damage in A549 cells and the DNA damage in CGN treated cells persist for a longer period of time and cannot be repaired. Our findings also indicate that natural compound CGN inhibits the cancer cell growth and induces G2/M phase arrest in A549 cells and significantly enhances the killing role of radiation.

In fact, cardenolides are known to exert cardiotonic effects by inhibiting the Na^+^/K^+^-ATPase. Because the binding of the sodium pump, they affect multiple signaling pathways and thus have a number of marked effects on tumor cell behavior. The cardenolide UNBS1450, which has been tested in a phase I clinical trial against NSCLC, can deactivate nuclear factor-κB (NF-κB)-mediated cytoprotective effects in human NSCLC. Nrf2 can crosstalk between the p53 and the other transcription factors NF-kB. It is possible that there are some relationships between NF-κB with the radiosensitivity. In addition, under normal condition, Nrf2 protein is bound to an inhibitor protein Kelch-like-ECH-associated protein (Keap1). Under oxidative stress conditions, the interaction between Nrf2 and Keap1 is disrupted leading to the translocation of Nrf2 into the nucleus resulting in transactivation of the target genes, which have ARE in their promoter regions [34, 35]. Maybe CGN can increase the levels of Keap1 and ultimately induce oxidative stress via disturbance of the redox status. Future studies will focus on the above two speculates to investigate the whole signaling pathway mechanism of the induction of the radiosensitivity in lung cancer cells after CGN treatment combined with irradiation.

Taken together, in this study we first report that pretreatment with CGN combined the irradiation decrease the survival of human lung cancer cells, compared to the irradiation only. CGN inhibits the up-regulation of the expression of antioxidant molecules in the irradiated lung cancer cells, which enhances the radiosensitivity of cancer cells but not to normal cells. The differential modulation of the intracellular redox state induced by CGN between A549 and BEAS-2B cells was associated with its selective radiosensitizing effect, suggesting that CGN is a potent radiosensitizer for lung cancer.

## MATERIALS AND METHODS

### Plant material

The stems of *C. gigantea* (Asclepiadaceae) were collected on August, 2012 in Hainan Province, P. R. China, and authenticated by Prof. Guo-Liang Zhang (Lanzhou University). A specimen (No. 2012081001) was stocked in the State Key Laboratory of Applied Organic Chemistry, Lanzhou University, P. R. China.

### Chemical extraction of cardenolides from *C. gigantea*

The air-dried and powdered stems and leaves (3.5 kg) were extracted with EtOH (3 × 10 L) at room temperature. The EtOH crude extract was evaporated in a vacuum to yield a residue, which was suspended in water then partitioned successively with petroleum ether, EtOAc and *n*-BuOH. The EtOAc fraction (168.0 g) was separated on a D101 macroporous resin column and eluted with a gradient mixture of H_2_O/EtOH (100:0, 70:30, 50:50, 20:80, 0:100 v/v) to give five fractions. Fr.50% H_2_O/EtOH (45.0 g) which exhibited the best activity against A549 cells was subjected to an MCI column chromatography (MeOH/H_2_O 100:0, 50:50, 60:40, 80:20, 90:10) to obtain four fractions (Fr.A-Fr.D). Fr.A (80% MeOH/H_2_O, 20.0 g) was further resolved on a silica gel column and eluted in a gradient of CHCl_3_/MeOH (100:1, 50:1, 10:1, 20:1, 10:1, 5:1, 1:1, 0:100, v:v) with increasing amounts of MeOH to give 5 subfractions (Fr.A.1-FrA.5). Fr.A.2 was subjected to passage over a Sephadex LH-20 column, eluting with MeOH, to yield compound 1 (5.8 mg). Fr.A.3 was submitted to a silica gel column, eluting with PE/EtOAc/MeOH (5:10:1, 5:20:1, 10:10:1, 20:10:1), and repeated this step three times to obtain pure 3 (< 2.0 mg) and 4 (< 2.0 mg). Fr.B (60% MeOH/H_2_O, 3.5 g) was separated over a RP-C18 column, eluting with MeOH/H_2_O (1:9, 3:7, 5:5, 6:4, 7:3) to afford 5 fractions (Fr.B.1-Fr.B.5), of which Fr.B.1 was composed entirely of 6 (16.0 mg). Fr.B.2 (1.5 g) was applied to a Sephadex LH-20 column, eluting with CHCl_3_/MeOH (1:1), to afford four fractions (Fr.B.2.1-Fr.B.2.4). Fr.B.2.1 was submitted to a silica gel column using CHCl_3_/MeOH (20:1, 15:1, v:v) for elution to obtain 2 fractions (Fr.B.2.1.1-Fr.B.2.1.2). Fr.B.2.1.1 was further purified by Sephadex LH-20 eluted with MeOH to afford 2 (2.3 mg) after semi-preparative HPLC (C18, MeOH/H_2_O, 0.95:1.05, v:v). Fr.B.2.1.2 was further purified by semi-preparative HPLC (C18, MeOH/H_2_O, 0.6:1.4, v:v) and obtained compound 7 (< 2.0 mg). Sephadex LH-20 eluted with MeOH to afford 10 (7.0 mg) after HPLC (MeOH/H_2_O, 0.95:1.05, v/v) by Fr.B.2.2. Fr.B.2.3 (5.5 g) was subjected to an MCI column chromatography (MeOH/H_2_O 100:0, 50:50, 60:40, 70:30, 80:20, 90:10, 100:0) to give four subfractions (Fr.B.2.3.1-Fr.B.2.3.4). After repeated silica gel column chromatography (EtOAc/MeOH, 20:1), Fr.B.2.3.2 (1.2 g) afforded compound 8 (11.3 mg). Fr.B.2.3.3 (1.8 g) was subjected to silica gel column chromatography, eluted with a gradient CHCl_3_/EtOAc (10:1) system to give compound 5 (1.5 mg). Fr.B.2.3.4 was purified by a Sephadex LH-20 column, eluting with MeOH, to afford compound 9 (2.2 mg).

### Compounds purity analysis

Compounds 1-10 were analyzed by HPLC to determine their purity. The analyses were performed on Waters 1525-2998 series HPLC system (C-18 column, X-bridge, 5 μm, 4.6 mm × 250 mm) at room temperature. The HPLC chromatograms of compounds 1-10 are included in the Supporting Information (Supplementary Table 2). All the tested compounds were dissolved in methanol. Methanol and water were used as mobile phase, and the flow rate was set at 1.0 mL/min. The maximal absorbance at the range of 210–400 nm was used as the detection wavelength.

### Cell culture

HeLa (human cervical carcinoma cell), A549 (human lung carcinoma cells), NCI-H460 (human lung carcinoma cells), NCI-H446 (human lung carcinoma cells), MRC5 (human normal lung fibroblast cells) and BEAS-2B (immortalized normal human bronchial epithelial cells) were obtained from the American Type Culture Collection (Manassas, VA, USA). HeLa, A549, 786-O and BEAS-2B cells were cultured in RPMI-1640 medium (Gibco, USA) supplemented with 100 U/mL penicillin, 100 μg/mL streptomycin and 10% fetal bovine serum (Gibco, USA) whereas human normal lung fibroblast MRC5 cells in Minimum Essential Medium (MEM), with 100 U/mL penicillin, 100 μg/mL streptomycin and 10% fetal bovine serum (Gibco, USA). Cell cultures were performed in a 5% CO_2_ atmosphere incubator at 37°C.

### Irradiation

X-ray irradiation was carried out by a Faxitron RX-650 facility (Faxitron Bioptics, USA), which was operated at 100 kVp and 5 mA at room temperature. The target of this instrument is wolframium (W). The dose rate was 0.451 Gy/min.

### MTT assay

Cells were seeded in 96-well plates with 5 × 10^3^ cells/well. After incubation for 12 h at 37°C, fresh medium (100 μL) containing various concentrations of the test compounds (the stocked concentration are 50–100 mM in sterile DMSO, −20°C) were added to the cells. Controls received an equal volume of DMSO. Following a 48 h incubation period, the medium was removed and the cells were further incubated for 4 h in the presence of 100 μL of 3-(4,5-dimethylthiazol-2-yl)-2,5-diphenyltetrazolium bromide (MTT) (the final concentration of MTT was 0.5 mg/mL). After dissolution of the resulting crystal formazan by the addition of DMSO (150 μL), the absorbance was measured at 570 nm using an automated microplate reader.

### Cell growth curve

Cells were seeded in 35 mm culture dishes with 2 × 10^4^ cells/dish overnight. Cells were treated with 1 μM CGN with a total volume of 2 mL for 24 h, and then were irradiated or sham-irradiated with 4 Gy X-rays. The mean number of cells per well was obtained four days later after irradiation from triplicate samples.

### Micronuclei assay

Micronucleus scoring was performed following the criteria established by Fenech. Cells were pretreated with or without CGN for 24 h followed by X-rays. 48 h later, 1 mL Carnoy's Fluid was added to every dish for 20–30 min and then rinsed with distilled water once. The air-dried cells were treated with acridine orange (0.01%) and then scored under fluorecent microscope (Zeiss, German). Five hundred viable cells were scored to determine the frequency of cells with 1, 2, 3 or 4 nuclei. Micronuclei were considered to be those structures morphologically identical to but smaller than the cell nucleus.

### γH2AX foci immunofluorescence

To detect γ-H2AX foci that form at the DSB sites, cells were grown on plastic coverslips as described previously [14]. The cells were reseeded at a density of 1 × 10^5^ cells in 35 mm cell culture dishes containing sterile coverslips, cells were then pretreated with/without CGN for 24 h and then irradiated. At 24 h post-irradiation, cells were fixed with 4% paraformaldehyde for 20 min. The fixed cells were treated with phosphate buffered saline (PBS) containing 1% fetal calf serum (FCS) and 0.5% TritonX-100 (TNBS) for 40 min. Cells were then treated the primary antibody for 90 min at room temperature. After primary antibody incubation, the cells were washed with PBS for approximately 5 min. After two washes with TNBS, the secondary antibody was applied for 30 min at room temperature. Digital image analysis was performed to determine the number of γH2AX radiation-induced foci. Quantification of foci per cell was done from images of 50-100 cells for time point from at least three independent experiments.

### Colony formation assay

Cells were treated with or without CGN (1 μM) for 24 h followed by irradiation. Cells were then trypsinized and scored. An appropriate number of cells from 80–10000 cells were seeded in the 60 mm diameter Petri dishes. Cells were irradiation 4 h later, and then cultured for 9-14 days. Fixed cells with alcohol and stained with crystal violet. Colonies containing more than 50 cells were identified as survivors under a stereomicroscope. Each experiment was performed in triplicate.

### Analysis of ROS levels

Cells (3 × 10^4^ cells/well) were seeded in 12-well plates. On the following day, the cells were exposed to CGN (0 or 1 μM) for 24 h, followed by X-ray irradiation (0 or 4 Gy) and then cultured for 24 h. After removal of the medium, cells were then washed with 1 × PBS solution. The ROS indicator DCFH-DA (10 μM) in fresh FBS-free medium was added to each well, and incubated continuously for 20–30 min at 37°C in the dark. The cells were visualized and photographed under fluorescent microscopy. The appearance of green fluorescence indicates the accumulation of ROS in cells. The fluorescence intensity were determined by Image J software.

### Cell transfection

SiRNA that targets to Nrf2 and its negative control were purchased from Genepharma (Shanghai, China). Nrf2 siRNA (sense: 5′-CCCGUUAGAUGACAAUTT-3′, antisense: 5′- AUUGUCUACAAACGGGTT-3′) was constructed as described [36]. Plated the A549 cells on the day before transfection at a confluence of 30%–50%. Transfection was performed with Lipofectamine 2000 (Invitrogen, USA) according to the manufacturer's instruction. The medium was changed with new culture medium 6 h post-transfection. The cells used in the following experiments were transfected for 24 h.

### Western blot

Cells were treated with 0 or 1 μM CGN for 24 h and then given 4 Gy X-rays irradiation. Cells were lysed in RIPA lysis and extraction buffer (25 mM Tris-HCl (pH 7.6), 150 mM NaCl, 1% NP-40, 1% sodium deoxycholate, 0.1% SDS) supplemented with protease inhibitor cocktail (Thermo Scientific) at the indicated time post irradiation. Protein concentration was determined using bicinchoninic acid protein assay (Pierce). Then cell lysates were resolved by SDS-PAGE under reducing conditions at a concentration of 30-50 μg protein of each sample per lane and then transferred to PVDF membranes (Millipore, Bedford, MA, USA). Blots were blocked with 5% bovine albumin in TBST for 2 h and then incubated overnight with primary antibodies (anti-Trx, anti-NQO-1, anti-HO-1, anti-TrxR1, and anti-Nrf2 1:500 dilution, Shenggong, Shanghai, China). They were then washed and incubated with a secondary peroxidase-conjugated antibody (1:2500 dilution, Abcam). Bound secondary antibody was detected using a chemiluminescence (ECL, Roche, Shanghai, China) system according to the manufacturer's protocol. To confirm equal protein loading per lane, the membranes were subsequently reprobed with a 1:2500 dilution of an anti-β-actin antibody (Shenggong) and developed as described above. The intensity of protein bands on the western blot image was quantified by Image J software.

### Flow cytometry analysis by propidium iodide staining

A549 cell were seeded in 35 mm culture dishes and incubated overnight, and then treated with sample or vehicle for 24 h, and then cells were irradiated or sham-irradiated, one day later the prepared cells were washed once with PBS, fixed in 70% ethanol at 4°C overnight, washed once with PBS, and stained with PI solution (20 μg/mL PI and 50 μg/mL RNase A in PBS) for 30 min in the dark. The samples were then analyzed by flow cytometry (Amnis, Imagestream, USA)

### Statistics

The statistical significance (*P* values) in mean values of two-sample comparison was determined with Student's *t*-test. A value of *P* < 0.05 was considered statistically significant (*) a value of *P* < 0.01 was considered extremely significant (**). Values shown on graphs represent the means ± SD of at least three independent repeated experiments.

## SUPPLEMENTARY MATERIALS FIGURES AND TABLES


