# Supplementary material for: Coroglaucigenin enhances the radiosensitivity of human lung cancer cells through Nrf2/ROS pathway
Source: Oncotarget. 2017 Mar 22;8(20):32807–20. doi: 10.18632/oncotarget.16454 (PMC5464829; doi:10.18632/oncotarget.16454)
Supplement: Supplementary file 1 [file oncotarget-08-32807-s001.pdf]

Coroglaucigenin enhances the radiosensitivity of human lung cancer cells through Nrf2/ROS pathway

Supplementary Materials

SUPPLEMENTARY INFORMATION

Compounds data

(19S)-3*β*,19-epoxy-2*α*,3*β*,14*β*-trihydroxy-19-methoxy-5*α*-card-20(22)-enolide (1)

white amorphous powder, ^{1}H NMR ($CDCl_3$, 400 MHz) 0.67 (H-1, dd, *J* = 12.7, 5.7 Hz), 2.58 (H-1, dd, *J* = 12.7, *J* = 9.5 Hz), 4.12 (H-2), 3.67 (H-3, t, *J* = 3.7 Hz), 2.21 (H-4, dd, *J* = 13.5, 10.2 Hz), 1.17 (H-4, dd, *J* = 13.5, 3.7, 1.6 Hz), 1.51 (H-5), 1.54 (H-6), 1.43 (H-6), 1.58 (H-8), 2.09 (H-15), 1.70 (H-15, dd, *J* = 13.3, 9.5 Hz), 2.15 (H-16), 1.86 (H-16), 2.82 (H-17, dd, *J* = 9.3, 5.5 Hz), 0.88 (H-18), 4.88 (H-19), 4.91 (H-21, dd, *J* = 18.4, 1.8 Hz), 5.03 (H-21, dd, *J* = 18.4, 1.8 Hz), 5.89 (H-19), 3.35 (OMe) ^{13}C NMR (100 MHz, $CDCl_3$) 35.7, 66.5, 70.3, 30.6, 40.2, 26.4, 27.4, 42.2, 45.5, 38.7, 22.7, 40.1, 49.3, 85.5, 33.1, 27.2, 50.8, 15.5, 90.8, 174.4, 73.4, 117.8, 177.3, 52.8. ESI-MS (m/z): $[M + H]^+$ 434.2; purity 94.62% ($MeOH/H_2O$ = 0.3/0.7, Rt = 8.294 min).

Uzarigenin (2)

white amorphous powder, ^{1}H NMR ($CDCl_3$, 400 MHz) 1.85 (H-1, m), 3.6 (H-3, m), 5.41 (t, *J* = 2.5), 2.78 (H-17, dd, *J* = 5.5, 9 Hz), 0.93(H-18), 1.02 (H-19), 5.87 (H-22). ^{13}C NMR (100 MHz, $CDCl_3$) 37.1, 31.4, 71.1, 37.9, 44.4, 28.5, 27.4, 41.7, 49.8, 35.7, 21.2, 39.9, 49.5, 85.5, 33.1, 26.9, 50.9, 15.8, 12.2, 174.5, 73.4, 117.7, 174.5.ESI-MS (m/z): $[M + H]^+$ 375.2; purity 95.72% ($MeOH/H_2O$ = 0.3/0.7, Rt = 8.438 min).

Digitoxingenin (3)

white amorphous powder, ^{13}C NMR (100 MHz, $CDCl_3$) 30.3, 27.9, 66.6, 33.3, 36.4, 26.9, 21.9, 41.3, 32.6, 35.5, 30.0, 74.8, 56.4, 85.8, 33.0, 27.9, 46.1, 9.4, 23.8, 177.1, 74.6, 117.0, 176.3. ESI-MS (m/z): $[M + H]^+$ 375.2; purity 92.76% ($MeOH/H_2O$ = 0.3/0.7, Rt = 8.519 min).

Corotoxigenin (4)

white amorphous powder, ^{1}H NMR ($CDCl_3$, 400 MHz) 1.85 (H-1, m), 3.6 (H-3, m), 5.41 (t, *J* = 2.5 Hz), 2.77 (H-17, dd, *J* = 5.5, 9.0 Hz), 1.57 (H-18), 10.03 (H-19), 4.95 (H-21, dd, *J* = 18.1, 1.2 Hz), 4.79 (H-21, dd, *J* = 18.4, 1.8 Hz), 5.89 (H-22). . ESI-MS (m/z): $[M + H]^+$ 375.2; purity 100% ($MeOH/H_2O$ = 0.3/0.7, Rt = 8.741 min).

Calotropagenin (5)

white amorphous powder, ^{1}H NMR (MeOD, 400 MHz) 0.93 (H-1α, m), 2.55 (H-1β, dd, *J* = 12.8 Hz, 4.4 Hz), 3.46 (H-2, H-3, m), 2.77 (H-17, m), 0.83 (H-18, s), 10.04 (H-19, s), 4.79 (dd, H-19α, *J* = 12.0 Hz), 4.94(dd, H-19β, *J* = 12.0 Hz), 5.88 (H-22, s). ESI-MS (m/z): $[M + H]^+$ 405.22; purity 94.57% ($MeOH/H_2O$ = 0.3/0.7, Rt = 9.792 min).

Coroglaucigenin (6)

white amorphous powder, ^{1}H NMR (MeOD, 400 MHz) 0.78 (H-1α, dt, *J* = 3.2, 17.6 Hz), 2.49 (H-1β, td, *J* = 3.6, 17.6 Hz), 1.86 (H-2α, m), 1.37 (H-2β, m), 3.64 (H-3, m), 1.68 (m, H-4α), 0.93 (m, H-4β), 2.14 (m, H-16α), 1.86(m, H-16β), 2.78 (H-17, dd, *J* = 5.5, 9 Hz), 0.94 (H-18, s), 3.81 (H-19α, *J* = 12.0 Hz), 3.67 (H-19β, *J* = 12.0 Hz), 4.88 (H-21, dd, *J* = 18.4 Hz, 1.2 Hz), 5.03 (H-21, dd, *J* = 18.4 Hz, 1.2 Hz),5.84 (H-22, s). ^{13}C NMR (100 MHz, MeOD) 32.6, 33.7, 71.7, 39.1, 46.0, 29.4, 28.7, 43.1, 52.2, 40.4, 24.0, 41.5, 51.2, 86.5, 32.5, 28.0, 51.5, 16.5, 59.2, 177.3, 75.4, 117.8, 178.6. ESI-MS (m/z): $[M + H]^+$ 391.24; purity 96.07% ($MeOH/H_2O$ = 0.3/0.7, Rt = 9.248 min).

Gomphoside (7)

white amorphous powder, ^{13}C NMR (100 MHz, MeOD) 44.3, 29.5, 72.8, 32.2, 47.5, 27.9, 29.5, 40.9, 50.5, 38.2, 26.7, 39.4, 50.6, 86.7, 38.6, 26.9, 51.3, 15.5, 20.7, 73.6, 117.2, 175.3, 175.3, 95.6, 91.1, 70.0, 33.1, 68.0, 22.6. ESI-MS (m/z): $[M + H]^+$ 519.29; purity 100% ($MeOH/H_2O$ = 0.4/0.6, Rt = 7.030 min).

Calotropin (8)

white amorphous powder, ^{13}C NMR (100 MHz, MeOD) 35.8, 68.2, 70.1, 33.0, 42.4, 27.3, 27.4, 43.3, 49.3, 52.3, 21.8, 39.2, 50.4, 84.9, 32.3, 26.7, 48.4, 15.5, 207.1, 174.3, 72.7, 117.9, 173.9, 95.5, 91.0, 71.7, 36.2, 68.0,

20.8. ESI-MS (m/z): $[M + H]^+$ 533.27; purity 96.57% ($MeOH/H_2O$ = 0.55/0.45, Rt = 29.073 min).

Calactin (9)

white amorphous powder, ^{13}C NMR (100 MHz, MeOD) 35.8, 69.6, 70.1, 33.0, 42.4, 27.3, 27.4, 43.3, 49.3, 52.3, 21.8, 39.2, 50.4, 84.9, 32.3, 26.7, 48.4, 15.5, 207.1, 174.3, 73.6, 117.9, 173.9, 93.9, 90.7, 71.5, 36.2, 66.6, 20.8. ESI-MS (m/z): $[M + H]^+$ 533.27; purity 95.32% ($MeOH/H_2O$ = 0.55/0.45, Rt = 29.068 min).

6′-*O*-(*E*-3,5-dimethoxy-4-hydroxycinnamoyl) desglucouzarin (10)

white amorphous powder, ^{13}C NMR (100 MHz, MeOD) 36.8, 29.9, 80.2, 33.4, 42.3, 28.0, 28.7, 40.8, 49.6, 35.4, 22.1, 38.4, 48.4, 86.2, 30.8, 28.0, 50.9, 16.3, 12.5, 178.5, 75.1, 117.7, 177.3, 102.9, 75.3, 78.2, 72.6, 75.2, 64.7, 167.0, 115.1, 146.7, 127.1, 116.9, 131.3, 161.5, 131.3, 116.9. ESI-MS (m/z): $[M + H]^+$ 743.36; purity 97.09 % ($MeOH/H_2O$ = 0.4/0.6, Rt = 32.878 min).

Supplementary Table 1: The sensitivity-enhancing ratio (SER)[a] at different doses (1, 2, 4, 6 Gy) in A549, NCI-H446 and NCI-H460 cells[b]

Dose (Gy)	SER (A549)	SER (NCI-H446)	SER (NCI-H460)
**1**	0.32	0.30	0.25
**2**	0.27	0.31	0.27
**4**	0.69	0.52	0.46
**6**	0.67	0.56	0.58

[a]The formula used to calculate the SER is: SER = ($SF_{\text{no CGN-treated}} - SF_{\text{CGN-treated}}$)/SF no CGN-treated.
[b]BEAS-2B did not show radiosensitivity when pretreated with CGN. Survival fractions of A549, NCI-H446 and NCI-H460 cells pretreated with or without CGN (1 μM) followed by 0, 1, 2, 4 and 6 Gy of X-ray irradiation were measured by colony score.

HPLC analyses

HPLC analyses were performed on Waters 1525-2998 series HPLC system (C-18 column, X-bridge, 5 μm, 4.6 mm × 250 mm; mobile phase; flow rate, 1.0 mL/min; UV wavelength, maximal absorbance at 210-400 nm; temperature, ambient).

Supplementary Table 2: HPLC Purity for Compounds 1-10

Sample	Peak time (min)	Purity (%)	Eluent (V/V)
**1**	8.294	94.62	0.3/0.7
**2**	8.438	95.72	0.3/0.7
**3**	8.519	92.76	0.3/0.7
**4**	8.741	100	0.3/0.7
**5**	9.792	94.57	0.3/0.7
**6**	9.248	96.07	0.3/0.7
**7**	7.030	100	0.4/0.6
**8**	29.073	96.57	0.55/0.45
**9**	29.068	95.32	0.55/0.45
**10**	32.878	97.09	0.4/0.6
